# The long-term effects of a polygenetic predisposition to general cognition on healthy cognitive ageing: evidence from the English Longitudinal Study of Ageing

**DOI:** 10.1017/S0033291721004827

**Published:** 2023-05

**Authors:** Olesya Ajnakina, Robin Murray, Andrew Steptoe, Dorina Cadar

**Affiliations:** 1 Department of Behavioural Science and Health, Institute of Epidemiology and Health Care, University College London, 1-19 Torrington Place, London, WC1E 7HB, UK; 2 Department of Biostatistics & Health Informatics, Institute of Psychiatry, Psychology and Neuroscience, King's College London, 16 De Crespigny Park, Camberwell, London, SE5 8AF, UK; 3 Department of Psychosis Studies, Institute of Psychiatry, Psychology and Neuroscience, King's College London, London, UK; 4 Department of Psychiatry, Experimental Biomedicine and Clinical Neuroscience, University of Palermo, Palermo, Italy

**Keywords:** APOE-*ɛ*4, cognition, genome-wide association studies, healthy ageing, polygenic score

## Abstract

**Background:**

As an accelerated cognitive decline frequently heralds onset of severe neuropathological disorders, understanding the source of individual differences in withstanding the onslaught of cognitive ageing may highlight how best cognitive abilities may be retained into advanced age.

**Methods:**

Using a population representative sample of 5088 adults aged •50 years from the English Longitudinal Study of Ageing, we investigated relationships of polygenic predisposition to general cognition with a rate of change in cognition during a 10-year follow-up period. Polygenic predisposition was measured with polygenic scores for general cognition (GC-PGS). Cognition was measured employing tests for verbal memory and semantic fluency.

**Results:**

The average baseline memory score was 11.1 (s.d. = 2.9) and executive function score was 21.5 (s.d. = 5.8). An increase in GC-PGS by one standard deviation (1-s.d.) was associated with a higher baseline verbal memory by an average 0.27 points (95% CI 0.19–0.34, *p* < 0.001). Similarly, 1-s.d. increase in GC-PGS was associated with a higher semantic fluency score at baseline in the entire sample (*β* = 0.45, 95% CI 0.27–0.64, *p* < 0.001). These associations were significant for women and men, and all age groups. Nonetheless, 1-s.d. increase in GC-PGS was not associated with decreases in verbal memory nor semantic fluency during follow-up in the entire sample, as well stratified models by sex and age.

**Conclusion:**

Although common genetic variants associated with general cognition additively are associated with a stable surplus to cognition in adults, a polygenic predisposition to general cognition is not associated with age-related cognitive decline during a 10-year follow-up.

## Introduction

Cognitive decline, including verbal memory and executive function, is among the most feared aspects of growing old, as it frequently heralds onset of dementia spectrum (Rajan, Wilson, Weuve, Barnes, & Evans, 2015) and other adverse health-related outcomes including mortality (Deary et al., 2009; Wilson, Beckett, Bienias, Evans, & Bennett, 2003). Although multiple factors may determine individual paths of cognitive decline, investigating the mechanisms underlying individual differences in withstanding the onslaught of cognitive ageing is likely to highlight how best cognitive abilities may be retained into advanced age (Deary, 2013).

General cognitive function has a heritability of >50–70% from adolescence through adulthood to older ages (Plomin & Deary, 2015; Tucker-Drob, Briley, & Harden, 2013). More specifically, heritability of general cognitive ability has been shown to have a linear increase from childhood (~40%) to adolescence (~50%) to adulthood (~60%) (Haworth et al., 2010; Plomin & Deary, 2015; Tucker-Drob et al., 2013). Genetic correlations among different cognitive domains are estimated to be >0.6, indicating that the same genes may be responsible for the heritability of these domains (Plomin & Kovas, 2005). More recent genomic studies of general cognition revealed that its genetic architecture is characterised by multiple common genetic markers spread across the entire genome where the identified genes additively explained 12–25% of variation in the trait of cognition (Davies et al., 2018; Savage et al., 2018). Building on the results from these genome-wide association studies (GWAS), the polygenic scores (PGS), which measure an individual genetic predisposition to a trait by combining the effects of many common genetic variants associated with it (Dudbridge, 2013), further demonstrated that individual differences in cognitive function are driven by thousands of common genetic markers scattered across the whole genome (Davies et al., 2018; Savage et al., 2018). Cumulatively, these studies demonstrated that a higher load of common genetic markers for general cognition are associated with better cognition.

Because genetic variants are determined randomly at conception and segregated independently of environmental influences, PGSs can be seen as unconfounded proxies for the life-time predisposition to general cognition. Consequently, one of possible ways to investigate the source of individual differences in normal, non-pathological cognitive ageing is by ascertaining whether a polygenetic propensity to general cognition is also associated with the rate of cognitive change in adults using the PGS approach. Although a decline in cognitive performance in adults is part of the normative ageing process (Wilson et al., 2002), having a higher load of common genetic markers associated with general cognition would be expected to associate with a lower rate of cognitive decline over time. However, this question has not been investigated.

Due to their vital functionality, verbal memory and semantic fluency cognitive domains are important predictors of clinically significant cognitive decline in healthy older adults (Masur, Sliwinski, Lipton, Blau, & Crystal, 1994). Indeed, verbal memory is necessary for storing and processing internally held information for use in guiding behaviours (Kofler et al., 2011). Whereas semantic fluency is arguably the most complex aspect of one's cognitive capacities. This is because semantic fluency is necessary for everyday living, such as attention, initiation, mental flexibility, organisation, abstract thinking, planning and problem-solving, all of which are required to implement behavioural responses appropriate to a constantly changing world (Kofler et al., 2011). Because these cognitive domains have varying functionalities, it is feasible that a polygenic predisposition to general cognition may be associated with a slower decline in these cognitive domains to varying degree. There is further evidence suggesting that age and gender differences exist in the cognitive ageing processes (Deary et al., 2009; Weber, Skirbekk, Freund, & Herlitz, 2014). For example, earlier twin studies highlighted that boys had a higher heritability for a verbal measure of cognitive ability compared to girls (Galsworthy, Dionne, Dale, & Plomin, 2000). More recent research in turn highlighted that this genetic propensity to a higher verbal measure of cognitive ability in men extends to a later part of life (Kępińska et al., 2020). However, the questions of the polygenic influence underlying these cognitive domains, and if their impact differs across genders and age groups, have not been investigated.

Despite some uncertainty about their ultimate clinical utility (Wray et al., 2013), PGSs can advance our knowledge of the genetic nature underlying rate of change in verbal memory and semantic fluency cognitive domains in adults. Therefore, we investigated whether a higher load of common genetic markers for general cognition associated with higher cognitive domains at baseline and with a deaccelerating decline in cognitive domains over the 10-year follow-up in the general population of adults. Assuming a variation in cognitive domains is a function of the degree of genetic liability to general cognition, we hypothesised that a PGS for general cognition would be significantly associated with a higher score in verbal memory and semantic fluency at baseline, and a lesser rate of decline in these cognitive domains during follow-up in health adults. Given age and gender differences in cognitive functions and cognitive ageing (Kępińska et al., 2020; Weber et al., 2014), we also investigated whether the potential relationships of a PGS for general cognition with cognition at baseline and during follow-up in healthy adults differed by age and gender.

## Methods

### Sample

We analysed data from the English Longitudinal Study of Ageing (ELSA), which is an ongoing large, multidisciplinary study of a nationally representative sample of the English adults aged ⩾50 years old (Steptoe, Breeze, Banks, & Nazroo, 2013). The ELSA study started in 2002–2003 (wave 1) with participants recruited from the Health Survey for England (HSE), who were then followed-up every 2 years. The sample is periodically refreshed with younger participants to ensure that the full age spectrum is maintained. Comparisons of the ELSA sample with the national census showed that the baseline sample was representative of the non-institutionalised general population aged ⩾50 living in the UK (Steptoe et al., 2013). For the present study, the baseline data were obtained from either wave 2 (2004–2005) for the core members who started at wave 1, or wave 4 (2008–2009) for the participants joining the study through refreshment samples; the included participants took part in the blood draws during home visits by a nurse. Follow-up data were ascertained from waves 4 (2008–2009) to wave 8 (2016–2017). We excluded participants with pathological causes of cognitive decline, such as clinical stroke (including self-reported stroke) or dementia at baseline and throughout the follow-up waves of data collection; we have further moved responders who died during the follow-up period. Our final analytic sample comprised 5088 responders. The full process of sample selection is depicted in online Supplementary Fig. S1. Compared to those who were excluded, the ELSA participants who were included in the study tended to be younger, had higher educational attainment and higher accumulated wealth; the latter group also included a lower proportion of people with long-standing health conditions, severe depressive symptoms and smokers compared to the respondents who were excluded from this study (online Supplementary Table S1). Ethical approval for each of the ELSA waves was granted by the National Research Ethics Service (London Multicentre Research Ethics Committee). All participants gave informed consent.

### Study variables

#### Cognition

To measure verbal memory, immediate and delayed verbal memory were assessed using a word-learning task, which entailed recalling as many out of 10 common words that were read out to them as possible immediately and after a short delay during which they completed other cognitive tests (Yin, Lassale, Steptoe, & Cadar, 2019). Following the protocol of previous studies (Fancourt & Steptoe, 2019), the results for immediate and delayed recall were then combined to give an overall verbal memory variable. Semantic fluency was measured with a verbal fluency test, where participants were asked to think of as many animal names as they could in one minute. The total number of animals named by participants was used as a measure of semantic fluency. Although this task primarily focused on semantic fluency, it combined various aspects of broader executive function including cognitive flexibility, processing speed, inhibitory control and verbal fluency. As the semantic fluency test was not administered at wave 6, there was an extended gap in the follow-up assessments of this cognitive domain during the follow-up. The distribution of these cognitive domains across all waves of data collection is provided in online Supplementary Table S2.

#### Covariates

Covariates include gender, year of birth and age; to capture the non-linear effects of ageing, we further included age^2^ as a covariate. Because the *ɛ*4 allele of the apolipoprotein E gene (*APOE*-*ɛ*4) has previously been associated with cognitive decline in normal ageing (Zhang & Pierce, 2014), we adjusted our analyses for *APOE*-*ɛ*4. Consistent with previous research (Zhang, Zhao, Xu, & Jiang, 2018), APOE-*ɛ*4 status was defined according to the absence or presence of *APOE* alleles (*ɛ*2/4, *ɛ*3/4 and *ɛ*4/4). Lastly, genetic ancestry [as was measured with principal components (see below)) was included among the covariates to account for any ancestry differences in genetic structures that could bias our results (Price et al., 2006).

### Genetic data

#### Quality control

The genome-wide genotyping was performed at University College London Genomics in 2013–2014 using the Illumina HumanOmni2.5 BeadChips (HumanOmni2.5-4v1, HumanOmni2.5-8v1.3) which measures ~2.5 million markers that capture the genomic variation down to 2.5% minor allele frequency. Using PLINK (Purcell et al., 2007) and VCFtools (Danecek et al., 2011), SNPs were excluded if they were non-autosomal, the minor allele frequency was <1%, if more than 2% of genotype data were missing and if the Hardy–Weinberg Equilibrium *p* value <10^−4^. Samples were removed based on call rate (<0.99), suspected non-European ancestry, heterozygosity and relatedness; participants were further removed if theier recorded sex phenotype was inconsistent with genetic sex. The summary of full quality control employed in the ELSA study is provided in online Supplementary Fig. S2 and Table S3. We employed the principal components analysis (Price et al., 2006) to identify those individuals who deviated from European ancestry (i.e. ethnic outliers). This set of analyses demonstrated the presence of ancestral admixture in the 65 individuals, who were subsequently removed; individuals who self-reported to be of non-European ethnicity were also removed. Using the updated sample, we calculated principal component, which then was used to adjust for possible population stratification in the association analyses (Price et al., 2006; Wang et al., 2009).

#### PGS analyses

PGS are calculated from the number of trait-associated alleles weighted by corresponding genotype effect sizes as estimated in a reference GWAS meta-analysis (Choi, Mak, & O'Reilly, 2020; Purcell et al., 2009). Therefore, the predictive accuracy of PGS is dependent on the strength of the GWAS summary statistics. Currently, there are two GWASs that provided summary statistics for general cognition (Davies et al., 2018; Savage et al., 2018). The first was carried out by Davies et al. that combined individuals of European ancestry from 56 population-based cohorts brought together by the Cohorts for Heart and Aging Research in Genomic Epidemiology (CHARGE), the Cognitive Genomics Consortium (COGENT) consortia and UK Biobank (UKB) (Davies et al., 2018); the second was carried out by Savage et al. that measured multiple dimensions of cognitive functioning across 14 cohorts including UKB and the COGENT (Savage et al., 2018). To ascertain which of these summary statistics to employ, we calculated an expected predictive accuracy of GC-PGS based on the results of each GWAS using Avengeme package implemented in R (http://www.rstudio.com/). Specifically, using information on sample sizes utilised in each GWAS and in the present study (*n*_ELSA_ = 6831, *n*_Davies et al. (2018)_ = 300 486, *n*_Savage et al. (2018)_ = 269 867), total number of independent markers in genotyping panel (*m*_Davies et al. (2018)_ = 1 348 174 and *m*_Savage et al. (2018)_ = 1 269 550), and lower and upper *p* values used to select markers into GC-PGS (*p*0, *p*1), our estimates showed that GC-PGS based on GWAS from Savage et al. (power = 0.504, *p* = 0.027) would have more power for the subsequent analyses compared to the predictive power of GC-PGS calculated based on summary statistics from Davies et al. (power = 0.386, *p* = 0.052). Using summary statistics provided by Savage et al., we calculated PGS for general cognition using PRSice based on genotyped data at different *p* value cut-offs (Euesden, Lewis, & O'Reilly, 2015). As it was highlighted that PGSs calculated based all available SNPs either explain the most amount of variation in a trait or are not significantly different than PGSs based on different *p* value thresholds, we utilised PGS at *p* value threshold of 1 (Ware et al., 2017). To aid the interpretability of the results, GC-PGS was standardised to a mean of 0 [standard deviation (s.d.) = 1].

### Statistical analysis

To assess the relationships of PGSs with verbal memory and semantic fluency and to estimate the mean change in each of these cognitive domains over a period of 10 years, we employed linear mixed-effect models (LMMs) with maximum likelihood estimation (Kristjansson, Kircher, & Webb, 2007). LMMs are flexible analytic tools for modelling correlated continuous data where correlations among values on continuous dependent variables arise from repeated measurements (West & Galecki, 2012). LMM models further maximise the use of longitudinal data by weighing estimates for missing data between waves and as such increase statistical power and precision (Mallinckrodt, Clark, & David, 2001). The maximum likelihood estimation aspect of LMMs seeks to find parameter values that make the model's predicted values most similar to the observed values (Martínez-Huertas, Olmos, & Ferrer, 2021). As LMMs have been developed to capture individual differences, while at the same time allowing generalisations across populations (Martínez-Huertas et al., 2021), we further captured random or stochastic variability in the data that comes from participants by estimating variance. Having considered linear, quadratic and cubic LMMs, Akaike Information Criterion and Bayesian Information Criterion (George, Seals, & Aban, 2014; Royston & Parmar, 2002) showed that the linear model was the most appropriate for our analyses. We first fitted a model adjusted for age, sex, year of birth and genetic ancestry as measured with 10 principal components (we referred to this model as Model 1). Next, to test for incremental predictive validity over *APOE*-*ɛ*4, which is a well-established risk factor for rapid cognitive decline, we fitted a second model which included all covariates from Model 1 in addition to *APOE*-*ɛ*4 (we referred to this model as Model 2). Interaction between GC-PGS and year of birth in the analyses of each cognitive domain was shown to be non-significant; thus, these interactions were not included in the models. For the age-stratified analyses, age groups were formed based on tertile results (i.e. 50–57, 58–65 and ⩾66 years). All analyses were weighted for non-response to requests for blood collections to wave 2 for the core members, or wave 4 for the participants who joined the study at wave 4 through the refreshment sample. Due to multiple testing, Bonferroni correction was applied (Benjamini & Hochberg, 1995), and significance was indicated within each model if *p* < 0.004 (*p* = 0.05/12). All association analyses were conducted in STATA release 14 (STATA Corp LP, USA).

## Results

### Sample characteristics

The baseline demographic and health characteristics of the total sample are presented in Table 1. The analytic sample comprised 5088 individuals with an average age for the entire cohort of 61.7 years old (s.d. = 7.4, median = 60, IQR = 56–67); of these, *n* = 1812 (35.6%) were aged 50–57 years old, *n* = 1745 (34.3%) were aged 58–65 years old, and *n* = 1531 (30.1%) were ⩾66 years old. Of the entire analytic sample, 24.8% (*n* = 1260) were *APOE*-*ɛ*4 carriers and 44.2% (*n* = 2250) were men. The average baseline memory score was 11.1 (s.d. = 2.9) and executive function score was 21.5 (s.d. = 5.8).

**Table 1.** Sample characteristics at baseline

Baseline sample characteristics	Total sample *n* = 5088
	*n* (%)/mean (s.d.)
Age at baseline (years)	61.7 (7.4)
Median (IQR)	60 (56–67)
Age groups	
50–57 years	1812 (35.6)
58–65 years	1745 (34.3)
⩾66 years	1531 (30.1)
Gender	
Female	2838 (55.8)
Male	2250 (44.2)
APOE-*ɛ*4 present	1260 (24.8)
Cognition	
Memory score	11.1 (2.9)
Executive function score	21.5 (5.8)

*APOE-ɛ4*, two *ɛ4* alleles of the apolipoprotein E gene; s.d., standard deviation.

### Change in cognition over the 10-year follow-up

**Fig. 1.**
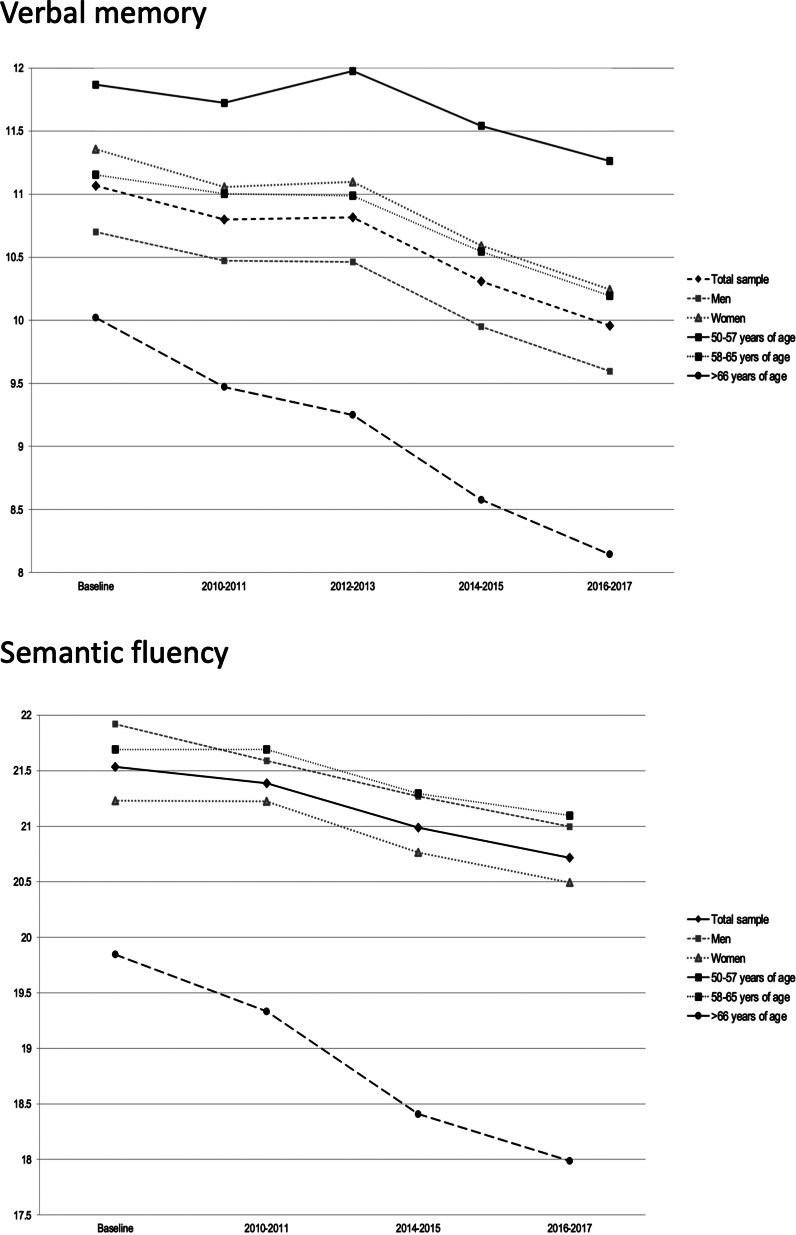
The average distribution of the verbal memory and semantic fluency cognitive domains across all waves of data collection over the 10-year follow up for the entire sample, different age groups and genders.

A significant decline in verbal memory (*β* = −0.13, 95% CI −0.15 to −0.12, *p* < 0.001) and semantic fluency (*β* = −0.31, 95% CI −0.36 to −0.25, *p* < 0.001) was observed during the follow-up period of 10 years (Fig. 1, online Supplementary Table S2). As further depicted in Fig. 1, a comparable decline in these cognitive domains was observed in men and women. While the decline in verbal memory and semantic fluency scores was comparable between the adults who were aged 50–65 years of age, the most striking decline was observed in adults who were aged ⩾66 years old.

### PGS for general cognition and verbal memory

An increase in GC-PGS by one standard deviation (1-s.d.) was associated with a higher baseline verbal memory by an average 0.27 points (Model 2: 95% CI 0.19–0.34, *p* < 0.001) (Table 2) indicating that a higher GC-PGS the adults had higher they scored on verbal memory test. This association was significant for women (*β*_model 2_ = 0.23, 95% CI 0.14–0.33, *p* < 0.001) and men (*β*_model 2_ = 0.32, 95% CI 0.21–0.42, *p* < 0.001). In the age-stratified analyses, 1-s.d. increase in GC-PGS was associated with the higher baseline verbal memory score by approximately 0.31 points in adults who were aged ⩾66 years old (Model 2: 95% CI 0.17–0.45, *p* = 0.001), 0.29 points in adults aged 50–57 years (Model 2: 95% CI 0.17–0.41, *p* < 0.001) and 0.19 points in adults who were aged 50–57 years older (Model 2: 95% CI 0.07–0.31, *p* = 0.001). Nonetheless, 1-s.d. increase in GC-PGS was not associated with decreases in verbal memory during follow-up in the entire sample, as well stratified models by sex and age.

**Table 2.** Associations between polygenic score for general cognition (GC-PGS) and longitudinal measure of verbal memory in older adults over the 10-year follow-up

Polygenic score for general cognition	Model 1			Model 2		
	*β*	95% CI	*p* Value	*β*	95% CI	*p* Value
Total sample						
Baseline	0.27	0.19–0.34	<0.001	0.27	0.19–0.34	<0.001
Rate of change	0.01	−0.01 to 0.02	0.325	0.01	−0.01 to 0.02	0.277
Variance^a^	0.19	0.18–0.20		0.19	0.17–0.20	
Women						
Baseline	0.23	0.13–0.33	<0.001	0.23	0.14–0.33	<0.001
Rate of change	0.01	−0.01 to 0.02	0.468	0.01	−0.01 to 0.02	0.404
Men						
Baseline	0.32	0.21–0.42	<0.001	0.32	0.21–0.42	<0.001
Rate of change	0.01	−0.01 to 0.02	0.548	0.01	−0.01 to 0.02	0.520
50–57 years						
Baseline	0.29	0.17–0.41	<0.001	0.29	0.17–0.41	<0.001
Rate of change	0.01	−0.01 to 0.02	0.396	0.01	−0.01 to 0.03	0.396
58–65years						
Baseline	0.19	0.07–0.31	0.001	0.29	0.11–0.35	0.002
Rate of change	0.02	−0.01 to 0.03	0.172	0.01	−0.01 to 0.03	0.190
⩾66 years						
Baseline	0.31	0.17–0.45	0.001	0.32	0.18–0.46	0.001
Rate of change	−0.004	−0.03 to 0.04	0.709	−0.003	−0.02 to 0.02	0.773

The presented *β* are standardised; CI, confidence intervals.

Model 1 is adjusted for baseline age, gender, year of birth and 10 principal components to account for any ancestry differences in genetic structures that could bias; Model 2 is adjusted for baseline age, gender, year of birth, APOE-*ɛ*4 and 10 principal components to account for any ancestry differences in genetic structures that could bias.

a The estimated variance captured random or stochastic variability in the data that comes from participants, here showing that there were significant individual differences in measure of verbal memory over time.

### PGS for general cognition and semantic fluency

A 1-s.d. increase in GC-PGS was associated with a higher semantic fluency score at baseline in the entire sample (*β*_model 2_ = 0.45, 95% CI 0.27–0.64, *p* < 0.001) and separately in women (*β*_model 2_ = 0.40, 95% CI 0.17–0.63, *p* = 0.001) and men (*β*_model 2_ = 0.54, 95% CI 0.24–0.83, *p* < 0.001) (Table 3). In the age-stratified analyses, 1-s.d. increase in GC-PGS was associated with a higher baseline semantic fluency score in adults who were aged 50–57 years old (*β*_model 2_ = 0.46, 95% CI 0.17–0.75, *p* = 0.002), and in adults aged 58–65 years (*β*_model 2_ = 0.54, 95% CI 0.22–0.86, *p* = 0.001). During the 10-year follow-up, 1-s.d. increase in GC-PGS was not associated with decreases in semantic fluency score in the entire sample and in stratified models.

**Table 3.** Associations between polygenic score general cognition (GC-PGS) and longitudinal measure of semantic fluency in older adults over the 10-year follow-up

Polygenic score for general cognition	Model 1			Model 2		
	*β*	95% CI	*p* Value	*β*	95% CI	*p* Value
Total sample						
Baseline	0.46	0.27–0.64	<0.001	0.45	0.27–0.64	<0.001
Rate of change	0.03	−0.03 to 0.08	0.331	0.03	−0.03 to 0.08	0.305
Variance^a^	2.38	2.24–2.52		2.37	2.24–2.52	
Women						
Baseline	0.40	0.16–0.62	0.001	0.40	0.17 to 0.63	0.001
Rate of change	0.04	−0.03 to 0.11	0.297	0.04	−0.03 to 0.11	0.286
Men						
Baseline	0.54	0.24–0.84	<0.001	0.54	0.24–0.83	<0.001
Rate of change	0.02	−0.07 to 0.11	0.646	0.02	−0.06 to 0.11	0.614
50–57 years						
Baseline	0.46	0.17–0.76	0.002	0.46	0.17–0.75	0.002
Rate of change	0.04	−0.04 to 0.12	0.338	0.04	−0.04 to 0.13	0.332
58–65years						
Baseline	0.54	0.22–0.86	0.001	0.54	0.22–0.86	0.001
Rate of change	0.002	−0.09 to 0.10	0.963	−0.003	−0.10 to 0.09	0.964
⩾66 years						
Baseline	0.38	0.03–0.73	0.032*	0.38	0.03–0.73	0.031*
Rate of change	0.02	−0.08 to 0.13	0.640	0.03	−0.07 to 0.13	0.555

The presented *β* are standardised; CI, confidence intervals.

Model 1 is adjusted for baseline age, gender, year of birth and 10 principal components to account for any ancestry differences in genetic structures that could bias; Model 2 is adjusted for baseline age, gender, year of birth, APOE-*ɛ*4 and 10 principal components to account for any ancestry differences in genetic structures that could bias.

a The estimated variance captured random or stochastic variability in the data that comes from participants, here showing that there were significant individual differences in measure of semantic fluency over time.

*These results did not survive Bonferroni correction for multiple testing (*p* = 0.05/12 = *p* < 0.004).

## Discussion

To our knowledge, this is the first study to have investigated the relationships of a polygenic predisposition for general cognition, as measured with the PGS approach, with individual differences in verbal memory and semantic fluency cognition domain at baseline and change in each of these cognitive domains over a period of 10 years independently of *APOE*-*ɛ*4 status in a large population-representative sample of adults.

Our results showed that a higher load of nominally associated loci for general cognition was associated with better verbal memory and semantic fluency in adults at baseline. All effect sizes for these significant effects ranged from modest (the standardised *β*s = 0.19 for verbal memory) to moderate (the standardised *β*s = 0.46 for semantic fluency). These results in turn highlight that individual differences in these cognitive domains are influenced by an individual load of common genetic markers associated with general cognition. It was previously argued that men aged 44–88 years old might differ from women of the similar age in their cognitive capacity (Finkel, Reynolds, Berg, & Pedersen, 2006), possibly due to differences in the prevalence of neuronal efficiency (Payton, 2009). Our results, however, demonstrated that these potential gender differences could not be explained by a polygenic predisposition to general cognition. In contrast to the results from cross-sectional samples that appeared to lend support for the notion that the heritability of cognitive functions decreases as adults age (Lee, Henry, Trollor, & Sachdev, 2010), we showed that the additive contribution of common genetic markers for general cognition were significantly associated with higher baseline verbal memory and semantic fluency scores across all age groups.

Although a significant decline in verbal memory and semantic fluency was observed over the follow-up time, in contrast to our hypothesis, common genetic variants associated with general cognition additively were not associated with a greater rate of decline in verbal memory and semantic fluency during the 10-year follow-up in adults from the general population. Employing PGS for educational attainment, neuroticism, Alzheimer's disease, major psychiatric disorders and physical health, Ritchie et al. showed that there were no statistically significantly associations between PGS and variation in cognitive change between ages 11 and 70–79 in the longitudinal Lothian Birth Cohort 1936 study (Ritchie et al., 2019). Our results extend these findings by showing that a PGS for general cognition also was not associated with cognitive domains in adults aged 50 years old and older. Our results are further aligned with other longitudinal studies which, having investigated determinants of the rate of cognitive decline on various cognitive domains over a 5-year (Reynolds, Finkel, Gatz, & Pedersen, 2002), 8-year (McGue & Christensen, 2002) and even a 16-year follow-up (Finkel, Reynolds, McArdle, Hamagami, & Pedersen, 2009), concluded the rate of cognitive decline in the general population is primarily driven by environmental factors.

The observed non-significant association between the genetic influences and cognitive change may also reflect attrition effects, which are unavoidable in longitudinal cohorts. Similarly, because the results presented in the study are based on longitudinal study with prospectively collected data, collider bias may have contributed to this finding (Arnold et al., 2021), which might have arisen from selection bias or attrition. Even though comparisons with the national census showed that the ELSA sample was representative of the non-institutionalised general population aged ⩾50 residing in the UK (Steptoe et al., 2013), to ensure we completely minimised any issues related to the selection bias we used inverse probability weighting in our models (Hernán, Hsu, & Brian, 2019). In terms of attrition, the proportion of missingness in the present study was comparable to many longitudinal cohorts (Ajnakina et al., 2021; Power & Elliott, 2006; Sonnega et al., 2014); we further imputed missing values using robust approaches (Ajnakina et al., 2021; Oba et al., 2003; Stekhoven & Bühlmann, 2012). Therefore, it is unlikely that attrition or selection bias influenced our results. It is nevertheless possible that only a subset of the genetic factors for general cognition may still have an impact on individual differences in non-pathological, age-associated change in semantic fluency, which, due to the nature of the PGS approach, might not have been captured in the present study. Therefore, further analyses, such as pathway-specific PGS analyses, genomic structural equation modelling and gene-set enrichment analyses, may be needed before we can draw more precise conclusions of the role common genetic loci may play in the rate of cognitive ageing.

### Methodological considerations

We analysed a large population-based cohort of nationally representative of older adults in England who were followed-up every 2 years. We further benefitted from the availability of repeated measures of cognition across a 10-year span. Our study included a relatively equal proportion of women and men from socio-economically diverse backgrounds. Confidence in these findings is strengthened using a LMM, which is an optimal way to describe the change in continuous dependent variables over time all the while taking the intra-individual and inter-individual variation into account. Instead of using composite scores for broad cognitive functions, we explored verbal memory and semantic fluency separately (Szoke et al., 2008).

Nonetheless, several methodological limitations warrant further reflection and discussion. Although PGSs have the potential to improve health outcomes through their eventual routine implementation as clinical biomarkers, the low generalisability of genetic studies across populations is noteworthy (Martin et al., 2019). This is because the construction of PGSs is mainly dependent on the availability of the summary statistics from GWASs, which are currently predominately based on European participants (Martin et al., 2019). It may be argued that the non-significant findings of the present study might have arisen due to the cognitive measures employed not being sufficiently sensitive to detect changes in cognitive status over the follow-up period of 10 years. However, having utilised the same sample, it was previously shown that *APOE*-*ɛ*4 (Kępińska et al., 2020) and loneliness in older people contributed to a decline in these cognitive domains over the 10-year follow-up period (Yin et al., 2019). This in turn demonstrates that the ELSA sample had the necessary sensitivity to detect changes in cognition over time.

## Conclusion

Drawing on a large, phenotypically well-defined sample of population-representative adults, we demonstrated that common genetic variants associated with general cognition additively are associated with a stable surplus to cognition in adults. However, our results showed that a polygenic predisposition to general cognition is not associated with age-related cognitive decline during a 10-year follow-up reiterating previous assertations that the rate of cognitive decline in the general population may be primarily driven by environmental factors.

## Supplementary material

For supplementary material accompanying this paper visit https://doi.org/10.1017/S0033291721004827. click here to view supplementary material
