# Supplementary material for: The long-term effects of a polygenetic predisposition to general cognition on healthy cognitive ageing: evidence from the English Longitudinal Study of Ageing
Source: Psychol Med. 2022 Feb 10;53(7):2852–60. doi: 10.1017/S0033291721004827 (PMC10235650; doi:10.1017/S0033291721004827)
Supplement: Supplementary file 1 [file S0033291721004827sup001.docx]

**Supplementary Figure 1.** Flowchart depicting the selection process of the analytical sample

**Supplementary Figure 2.** Depicts every step of quality control and assurance that was undertaken in preparation of the genetic data for the analyses in the ELSA study


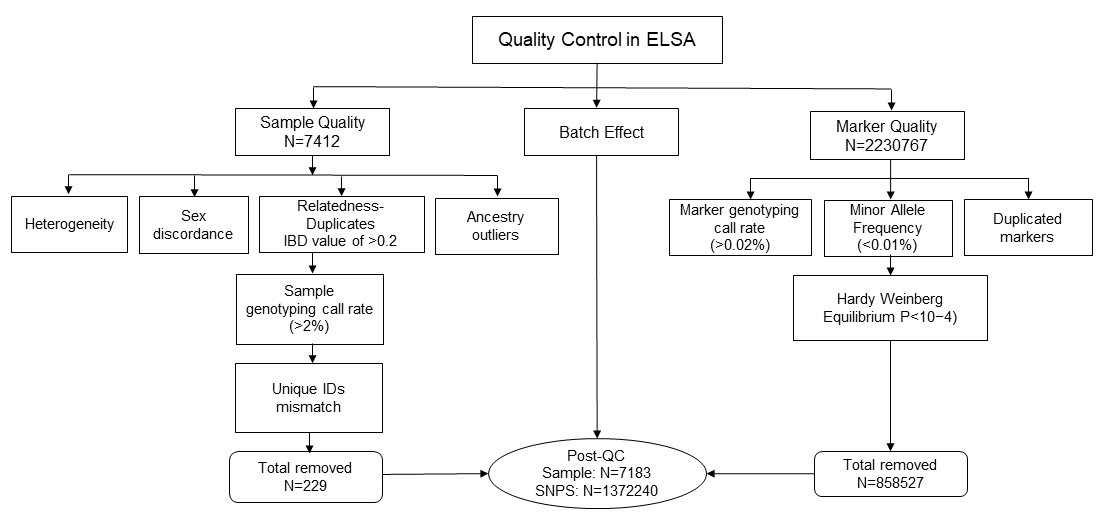


**Supplementary Table 1.** Comparisons between the ELSA participants who were included in the analyses and those who were excluded

|  |  |  | **Excluded (N=7854)** | **Included (N=5088)** |  |
| --- | --- | --- | --- | --- | --- |
|  |  |  | Mean (SD) / N(%) | Mean (SD) / N(%) | Statistics |
| *Socio-demographic characteristics* | |  |  |  |  |
|  | Age (years) |  | 65.8 (11.7) | 61.7 (7.4) | t=22.48, df=12940, p<0.001 |
|  | Gender (male) |  | 3515 (44.7) | 2250 (44.2) | x^2^=0.35, df=1, p=0.552 |
|  | Education (years) |  | 13.3 (3.8) | 14.1 (3.8) | t=-10.76, df=11892, p<0.001 |
|  | Accumulated wealth |  |  |  | x^2^=257.92, df=2, p<0.001 |
|  | High |  | 2868 (38.7) | 1263 (25.5) |  |
|  | Intermediate |  | 2398 (32.4) | 1744 (35.2) |  |
|  | Low |  | 2143 (38.9) | 1944 (39.3) |  |
|  |  |  |  |  |  |
| *Comorbid health issues* | |  |  |  |  |
|  | Limiting health conditions |  | 3110 (40.1) | 1329 (26.1) | x^2^=265.42, df=1, p<0.001 |
|  | Depression (score ≥4) |  | 970 (18.4) | 463 (11.8) | x^2^=74.02, df=1, p<0.001 |
|  |  |  |  |  |  |
| *Behavioural outcomes* | |  |  |  |  |
|  | Currently a smoker |  | 1361 (17.6) | 672 (13.2) | x^2^=43.20, df=1, p<0.001 |

**Supplementary Table 2.** Distribution of the cognitive domains for each wave of data collection for all ELSA participants included in this study

| **Cognitive domain and Age** |  | **Baseline** | **Wave 5:**  **2010-2011** | **Wave 6: 2012-2013** | **Wave 7:**  **2014-2015** | **Wave 8: 2016-2017** |  |
| --- | --- | --- | --- | --- | --- | --- | --- |
|  |  | Mean (SD) | Mean (SD) | Mean (SD) | Mean (SD) | Mean (SD) | Group differences |
|  |  |  |  |  |  |  |  |
| ***Verbal memory*** |  | 11.1 (2.9) | 10.8 (3.3) | 10.8 (3.4) | 10.3 (3.6) | 10.0 (3.8) | F=88.14, p<0.001 |
| Media (IQR) |  | 11 (9-13) | 11 (9-13) | 11 (9-13) | 10 (8-13) | 10 (8-12) |  |
|  |  |  |  |  |  |  |  |
| ***Semantic fluency*** |  | 21.5 (5.8) | 21.4 (6.3) | - | 21.0 (6.7) | 20.7 (6.8) | F=17.06, p<0.001 |
| Media (IQR) |  | 21 (18-25) | 21 (17-25) | - | 21 (17-25) | 21 (16-25) |  |
|  |  |  |  |  |  |  |  |
| ***Age (years)*** |  | 61.7 (7.4) | 66.7 (10.0) | 68.5 (7.5) | 70.7 (7.9) | 72.7 (7.9) |  |
| Media (IQR) |  | 60 (56-67) | 65 (61-72) | 67 (63-74) | 69 (65-76) | 71 (66-77) |  |

SD, standard deviation; ELSA, English Longitudinal Study of Ageing

**Supplementary Table 3.** An overview of the summary of full QC procedure employed in the ELSA study and how many variants and/or participants were lost at each step.

| Quality Control steps in ELSA | | | |
| --- | --- | --- | --- |
| *Lost due to SNP-based QC* | | *n* | % |
|  | Missing SNPs (0.02) | 41614 | 1.87 |
|  | Autosomal SNPs | 48578 | 2.18 |
|  | MAF 0.01 | 759972 | 34.07 |
|  | *Update rsids | 2284 | 0.10 |
|  | HWE (0.0001) | 6079 | 0.27 |
|  |  |  |  |
|  | *Total removed* | *858527* | *38.49* |
|  | *Total remaining* | *1372240* | *61.51* |
| *Lost due to Individual-based QC* | |  |  |
|  | Missingness (0.02) | 39 | 0.53 |
|  | Heterogeneity | 76 | 1.03 |
|  | Sex discordance | 5 | 0.07 |
|  | Ancestry outliers | 64 | 0.86 |
|  | Relatedness/Duplicates | 5 | 0.07 |
|  | Unique IDs are not present | 41 | 0.50 |
|  |  |  |  |
|  | *Total removed* | *229* | *3.09* |
|  | *Total remaining* | *7183* | *96.91* |

HWE, Hardy-Weinberg equilibrium; MAF, minor allele frequency; SNP, single nucleotide polymorphisms

***** To ensure a large overlap between the GWAS summary statistics (i.e., base file) and the ELSA (i.e., target) data, we have converted all present platform specific ids (i.e., kgps) to rsids. However, not all kgps were able to be successfully updated; those and single-nucleotide polymorphism (SNPs) for which the kgps were not updated were removed.
